# Mapping of population disparities in the cholangiocarcinoma urinary metabolome

**DOI:** 10.1038/s41598-021-00530-0

**Published:** 2021-10-28

**Authors:** Munirah Alsaleh, Zoe Leftley, Thomas O’Connor, Thomas Hughes, Thomas A. Barbera, Larry K. Koomson, Abigail Zabron, Helen Reeves, Matthew Cramp, Stephen D. Ryder, Shaun Greer, Martin Prince, Paiboon Sithithaworn, Narong Khuntikeo, Watcharin Loilome, Puangrat Yongvanit, I. Jane Cox, Roger Williams, Christopher A. Wadsworth, Elaine Holmes, Kathryn Nash, Ross Andrews, Simon D. Taylor-Robinson

**Affiliations:** 1 Department of Metabolism, Digestion and Reproduction, Imperial College London, London, W2 INY UK; 2 Northern Institute for Cancer Research, Paul O’Gorman Building, Medical School, University of Newcastle, Framlington Place, Newcastle upon Tyne, NE2 4HH UK; 3 Liver Unit, Derriford Hospital, Derriford Road, Crownhill, Plymouth, PL6 8DH Devon UK; 4 NIHR Nottingham Biomedical Research Centre, Nottingham University Hospitals NHS Trust and Nottingham Digestive Diseases Centre, University of Nottingham, Queen’s Medical Centre, Nottingham, NG7 2UH UK; 5 Centre for Hepatology, Manchester Royal Infirmary, Oxford Road, Manchester, M13 9WL UK; 6 Cholangiocarcinoma Research Centre, Faculty of Medicine, Khon Kaen University, Khon Kaen, 40002 Thailand; 7 Foundation for Liver Research, The Roger Williams Institute of Hepatology, 111 Coldharbour Lane, London, SE5 9NT UK; 8 Faculty of Life Sciences and Medicine, King’s College London, London, UK; 9 Liver Unit, Southampton General Hospital, Tremona Rd, Southampton, SO16 6YD Hampshire UK

**Keywords:** Biomarkers, Diseases, Gastroenterology, Medical research

## Abstract

Phenotypic diversity in urinary metabolomes of different geographical populations has been recognized recently. In this study, urinary metabolic signatures from Western (United Kingdom) and South-East Asian (Thai) cholangiocarcinoma patients were characterized to understand spectral variability due to host carcinogenic processes and/or exogenous differences (nutritional, environmental and pharmaceutical). Urinary liquid chromatography mass spectroscopy (LC–MS) spectral profiles from Thai (healthy = 20 and cholangiocarcinoma = 14) and UK cohorts (healthy = 22 and cholangiocarcinoma = 10) were obtained and modelled using chemometric data analysis. Healthy metabolome disparities between the two distinct populations were primarily related to differences in dietary practices and body composition. Metabolites excreted due to drug treatment were dominant in urine specimens from cholangiocarcinoma patients, particularly in Western individuals. Urine from participants with sporadic (UK) cholangiocarcinoma contained greater levels of a nucleotide metabolite (uridine/pseudouridine). Higher relative concentrations of 7-methylguanine were observed in urine specimens from Thai cholangiocarcinoma patients. The urinary excretion of hippurate and methyladenine (gut microbial-host co-metabolites) showed a similar pattern of lower levels in patients with malignant biliary tumours from both countries. Intrinsic (body weight and body composition) and extrinsic (xenobiotic metabolism) factors were the main causes of disparities between the two populations. Regardless of the underlying aetiology, biological perturbations associated with cholangiocarcinoma urine metabolome signatures appeared to be influenced by gut microbial community metabolism. Dysregulation in nucleotide metabolism was associated with sporadic cholangiocarcinoma, possibly indicating differences in mitochondrial energy production pathways between cholangiocarcinoma tumour subtypes. Mapping population-specific metabolic disparities may aid in interpretation of disease processes and identification of candidate biomarkers.

## Introduction

Metabolic signatures are largely the results of interactions among genetic, environmental (including dietary) and gut microbial factors^1^. The unique characteristics of Isaan people from North-East Thailand, where eating raw, or partially cooked and/or fermented cyprinid fish from river systems which flow into the Mekong River leads to the frequent development of *Opisthorchis viverrini* liver fluke-associated cholangiocarcinoma (CCA), may lead to population-specific metabolic patterns in the urinary profile of both heathy and diseased individuals^2^. In a study applying nuclear magnetic resonance (NMR)-based metabolomics to a large-scale dataset (n = 4630, from China, Japan, UK and USA), geographic differences were found to contribute greatly to metabolic phenotype variation in urine, and were of greater magnitude than gender-related metabolic differences^3^. Interestingly, the metabolic signature across Western populations (American and British) were not easily differentiated, whereas the metabolic phenotypes of East Asian and Western populations were significantly differentiated (*p* < 10), owing to differences in metabolites of predominantly dietary and microbial origin^3^.

Few studies, mostly in vitro, examined CCA tumorigenesis mechanisms in sporadic CCA, compared to liver fluke-associated malignancy. Similarities and differences in CCA clinicopathological^4^, histopathological^5^, immunohistochemical^6^ and gene expression^7^ profiles between sporadic and *O.viverrini*-related tumors have been noted, but it is difficult to compare and interpret findings from these studies as anatomical tumor sites were different—the Japanese studies being focused on intrahepatic tumors only. From current evidence, bile duct tumors exhibit different biological behaviors that can affect pathogenesis and outcome, depending on their anatomical location^8^. Additionally, although CCA risk in Japanese individuals remains unclear, CCA incidence in Japan is relatively greater compared to Western counties and others Asian counties, such as Singapore and Philippines^9^. The higher rates of CCA incidence in Japan could be attributed to etiological factors such as hepatolithiasis (intrahepatic gallstones) and infection with viral hepatitis C (30% of CCA patients are positive for HCV-Ab in Japan)^10^.

Intestinal goblet cell phenotypes (antigen 17NM-positive) were found to be more frequently expressed in tissues from fluke-infested bile ducts, compared to sporadic cases from an Australian cohort^6^. In a more recent publication by the same Australian team, adenocarcinomas of the gallbladder also expressed a similar profile, 17NM was expressed significantly more by gallbladder (59%) and fluke-associated cancers (45%) than by sporadic CCA (17%), suggesting a chronic inflammatory background in carcinoma of the gallbladder and bile ducts^11^.

The main focus of our study was to compare the global urinary metabotype from a Thai population with *Opisthorchis*-associated CCA, to a Western patient population where CCA is sporadic. Another aim was to characterize the baseline healthy metabolome of the two distinct populations.

## Methodology

### Ethics and sample collection

Ethical approval was obtained from Imperial College London REC, London, UK (REC Reference 09/H0712/82) and prior written, informed consent was obtained from each participant. The study was conducted according to the principles set out in the 1975 Declaration of Helsinki.

#### Thailand

Study samples were collected from Isaan peoples, from North-Eastern region of Thailand. Raw, partially cooked and/or fermented fish dishes which are likely to contain the *O. viverrini* parasite are distinctive to their cultural cuisine. Patients with *O. viverrini*-associated CCA were recruited from the inpatient population in Khon Kaen Hospital, Khon Kaen, Thailand. Malignant strictures were diagnosed by computed tomography (CT) or magnetic resonance imaging (MRI) and further confirmed by histology at surgical operation. Spot urine samples were collected from each participant prior to the patient undergoing any treatment. The healthy participants were collected from the “CASCAP” field screening program in the endemic North-Eastern region^12^.

#### UK

Urine samples from CCA patients were collected from participating UK liver centers in London, Manchester, Newcastle, Nottingham, Plymouth and Southampton, and transported frozen to the Hepatology Biobank at St. Mary’s Hospital, London, UK. Potential participants were identified and recruited by their clinician from the in-patient or out-patient populations. None of the CCA patients from this UK cohort had an identifiable cause for their CCA (sporadic CCA). Healthy volunteers were sought from amongst visitors to the hospital, staff and students. After participants provided consent, they were assessed at baseline for demographic data, medical history, drug history and dietary history. These data have previously been included in a larger study characterising the CCA profile of a UK population^13^.

### Sample analysis

Samples from both populations were simultaneously analyzed using liquid chromatography mass spectroscopy (LC–MS) metabolic profiling to allow comparability. A subset of 66 raw spectral profiles were selected and pre-processed for chemometric data analysis.

### Chromatographic conditions

The samples spectra were acquired using an ACQUITY ultra performance liquid chromatography,(UPLC) system (Waters Ltd. Elstree, U.K.), coupled to a LCT Premier mass spectrometer (Waters MS Technologies, Ltd., Manchester, U.K.)^13^. Reverse phase (RP)-UPLC-MS was performed with electrospray ionisation (ESI) in both positive and negative modes^13^. The conditions were optimized using quality control (QC) samples in terms of peak shape, reproducibility and retention time^13^.

### Tandem mass spectrometry

Tandem mass spectrometry (MS/MS) analysis was performed using a quadrupole time-of-flight (TOF) Premier instrument (Waters MS Technologies, Manchester, UK)^13^. Collision-induced dissociation (CID) experiments of the QC sample were performed for structural elucidation of detected ions in each ionisation mode^13^. This was conducted subsequent to the original profiling run to save time and limit analytical variations in retention time and performance that can occur when returning to the instrument for CID analysis^13^. Two complementary tandem mass spectroscopy (MS/MS) acquisition modes were used to ensure sufficient MS/MS coverage of ions of interest, data-dependent acquisition (DDA) and acquisition with no precursor ion selection, or data-independent acquisition (MS^E^)^13^. The DDA experiment was set to switch automatically from the MS to MS/MS mode using data-dependent criteria^13^. It triggered MS/MS on the most abundant ions in each MS scan and provided fragments specifically attributed to the precursor ion^13^. In MS^E^ mode, eluting peaks were subjected to both high and low collision energies in the collision cell of the mass spectrometer, with no prior precursor ion selection^13^.

### Metabolite assignment verification

The molecular mass, retention time and fragmentation spectrum of the discriminant features were compared against on-line spectral libraries such HMDB (www.hmdb.ca)^5^ and METLIN (https://metlin.scripps.edu)^6^. Metabolites were classified as either:

- identified compounds confirmed with an authentic standard;
- putatively annotated compounds (such as those based upon fragmentation pattern and/or spectral similarity with spectral databases);
- putatively identified to match a certain chemical class (such as those based on spectral similarity to known compounds of a chemical class); or
- as unknown compounds.

### Pre-processing

The raw LC–MS data files were converted to CDV format by MassLynx version 4.1 application manager (Waters Corporation, Milford, U.S.A.) and then imported into R Project version 3.1.0 (The R Foundation for Statistical Computing, 2014) for pre-processing using XCMS package version 2.14. (Bioconductor). Computational scripts written in-house were applied to: (1) filter and identify peaks; (2) correct for retention time drift; (3) match peaks across samples; and (4) fill in missing peaks.

### Statistical analysis

SIMCA-P + version 13.0.2 (Umetrics, Umeå, Sweden) was used for multivariate statistical analysis of the processed data. MetaboAnalyst 3.0 was used for univariate analysis^13^.

### Ethics approval

Ethical approval was obtainned from Imperial College London REC, London, UK (REC Reference 09/H0712/82).

### Consent to participate

Prior written, informed consent was obtained from each participant.

## Results

### Demographics and cohort description

The characteristics of the study population (*n*= 66) are described in Table 1. The participants’ age ranged from 27 to 78 years with an average age over 60 years in patients with CCA, whereas healthy controls were younger, particularly in the UK cohort (mean age= 34 years). The gender distribution was in favor of males in the Thai group (healthy= 65% and CCA= 71%), but was relativity equally distributed among the UK group (healthy= 45.4% and CCA= 44.4%). The anatomical location of the tumors in the biliary tree varied between the two disease groups. Patients from Thailand presented predominantly with intrahepatic bile duct lesions, whereas lesions occurring in the perihilar region were more common in the participants from United Kingdom.

**Table 1:** Demographics of study population.

Characteristic	Thailand		United Kingdom	
	Healthy	CCA	Healthy	CCA
Participants, *n*	20	14	22	10
Age, mean, (range)	58 (29–74)	60 (29–77)	34 (24–58)	69 (57–78)
Male, %	65.0	71.4	45.4	44.4
**Tumour location**				
*Intrahepatic*	–	6	–	1
*Perihilar*	–	2	–	4
*Distal*	–	1	–	2
*Gallbladder*	–	3	–	–
*Unspecified*	–	2	–	3

### Drug exposure

Urine samples from all the cancer patients were dominated by metabolic features related to drug exposure, and this was primarily seen in CCA cases from the United Kingdom (Table 2). Correlation analysis was used to select and exclude spectral features related to drug intake.

**Table 2:** Top 10 discriminant features between cholangiocarcinoma cases from Thailand and United Kingdom.

*m/z*	RT	Compound	Adduct	Class	VIP	Trend
152.071	2.46	Paracetamol	M + H	analgesic	11.3	UK
140.017	2.64	Paracetamol cysteine	fragment	analgesic metabolite	10.1	UK
271.076	2.64	Paracetamol cysteine	M + H	analgesic	8.7	UK
208.043	2.64	Paracetamol cysteine	fragment	analgesic metabolite	8.4	UK
152.071	2.97	Paracetamol	Isomer	analgesic	7.3	UK
396.044	3.87	Ceftriaxone	fragment	antibiotic metabolite	11.5	Thai
555.054	3.87	Ceftriaxone	M + H	antibiotic	11.2	Thai
332.14	4.03	Ciprofloxacin	M + H	antibiotic	12.5	UK
333.146	4.03	Ciprofloxacin	isotope	antibiotic metabolite	8.4	UK
443.218	5.13	Metaxolone	2 M + H	analgesic/muscle relaxant	9.8	UK
326.087	2.46	Paracetamol glucuronide	M-H	analgesic	4.6	UK
653.182	2.46	Paracetamol glucuronide	2 M-H	analgesic metabolite	6.4	UK
246.007	2.53	Unknown			6.0	UK
299.035	2.91	Unknown			4.1	UK
260.022	3.06	Unknown			7.9	UK
311.07	3.53	Unknown			5.4	UK
623.147	3.53	Unknown			5.7	UK
276.001	3.66	Diclofenac/Voltaren	M-H2O-H	NSAID^†^	8.3	UK
410.081	4.79	Sulfociprofloxacin	M-H	antibiotic	9.6	UK
246.949	5.22	Unknown			18.8	UK

^†^Non-steroidal anti-inflammatory drug;

m/z = mass–charge ratio; RT = retention time; VIP = variable importance in projection score.

### General overview

**Figure 1:**
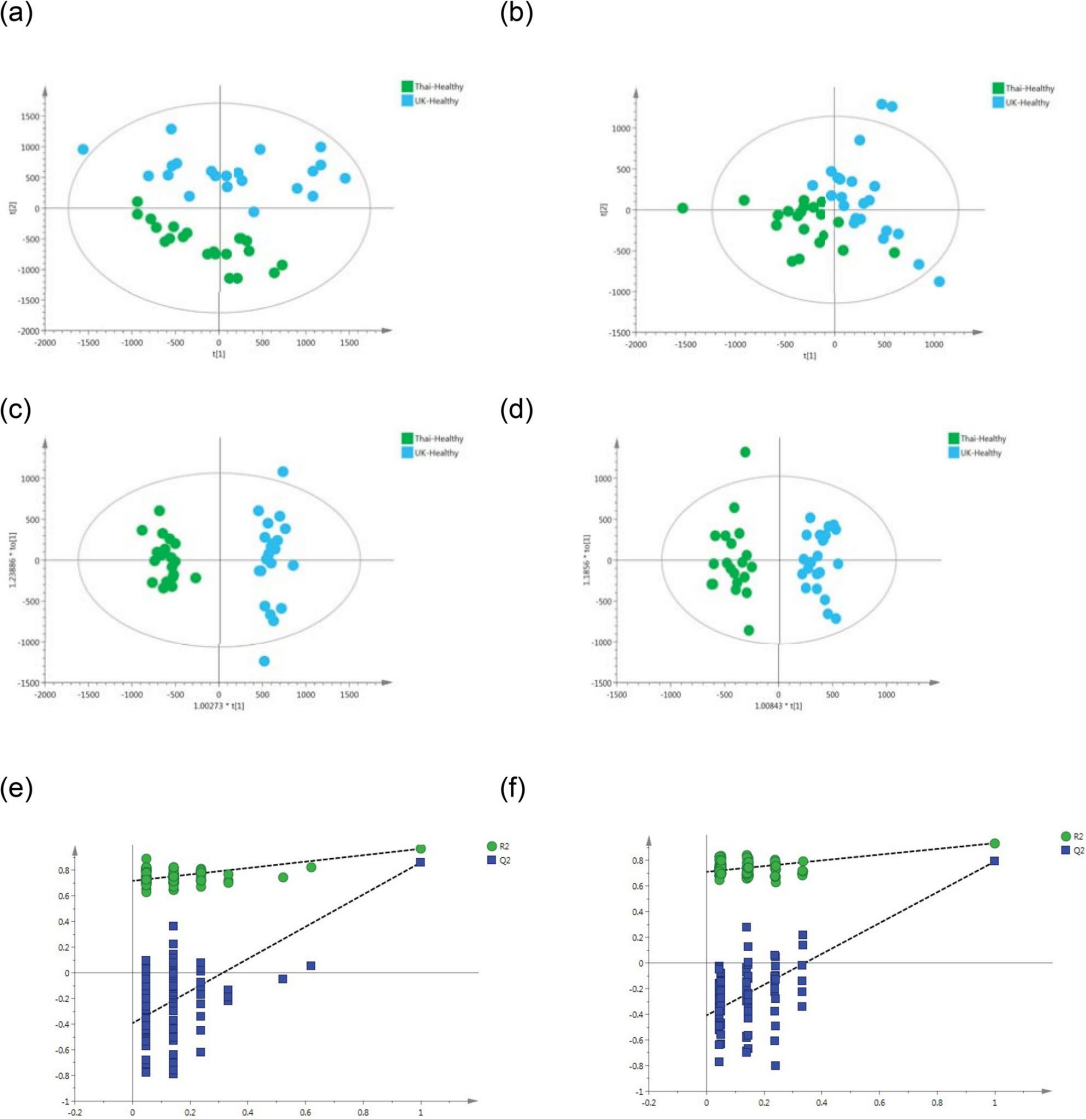
PCA scores plots for (**a**) positive and (**b**) negative ion mode data of healthy participants from Thailand and United Kingdom. OPLS-DA scores plots showing group separation for both (**c**) positive and (**d**) negative ion mode data and corresponding permutation tests for (**e**) positive and (**f**) negative ion mode data. (**a**) R2X = 30% and Q2X = 14% from 2 PC model; (**b**) R2X = 26% and Q2X =  − 4% from 2 PC model; (**c**) R2Y = 95%, R2X = 21% and Q2Y = 87%, from one predictive and one orthogonal components model; (**d**) R2Y = 93%, R2X = 17% and Q2Y = 79%, from one predictive and one orthogonal components model; (**e**) Permutation test—positive mode CV-ANOVA =  < 0.001; (**f**) Permutation test—negative mode CV-ANOVA =  < 0.001.

All possible comparisons using the spectral profiles from the dataset yielded statistically significant models. The healthy metabolome from the two populations was highly distinguishable, electropspray ionisation positive mode, ESI+ = R^2^ Y=95%, R^2^ X=21% and Q^2^ Y=87%; and electrospray ionisation negative mode, ESI– = R^2^ Y=93%, R^2^ X= 17% and Q^2^ Y=79% (Figure 1, Table 3).

**Table 3:** Altered metabolites between healthy participants from United Kingdom (*n* = 22) and Thailand (*n* = 20).

*m/z*	RT	Tentative assignment	Adduct	VIP	Trend in UK-Healthy	p-value^‡^	FC	Identification^†^
144.102	0.58	Proline betaine	M + H	17	↑	< 0.001	4.86	b
160.133	0.70	Unknown		5.1	↑	< 0.001	3.63	d
229.155	0.99	Isoleucyl proline	M + H	10.4	↓	< 0.001	1.81	b
137.046	1.11	Hypoxanthine	M + H	6.1	↓	0.003	1.55	b
166.073	1.17	7-Methylguanine	M + H	4.1	↑	NS	1.35	b
167.057	2.38	7-Methylxanthine	M + H	5.3	↑	< 0.001	5.84	b
180.088	2.88	Fructosamine	M + H	4.3	↓	0.006	1.44	b
126.092	3.01	Unknown		6.4	↓	< 0.001	2.58	d
181.073	3.18	Paraxanthine	M + H	4.7	↑	NS	2.01	b
105.033	3.82	Hippurate	M + H	3.9	↑	NS	1.2	a
287.207	4.93	Acylcarnitine (C8:1)	M + H	7	↑	< 0.001	7.74	b
312.217	5.17	2-trans,4-cis-Decadienoylcarnitine	M + H	5.2	↑	NS	3.62	b
310.202	5.35	Acylcarnitine (C10:3)	M + H	11.2	↑	< 0.001	6.81	b
314.233	5.78	Decenoylcarnitine (C10:1)	M + H	5.3	↑	0.021	2.18	b
243.061	0.85	Uridine/pseudouridine	M-H	3.8	↓	< 0.001	− 1.58	b
191.018	0.96	Isocitrate	M-H	8.0	↑	NS	1.25	b
191.018	1.07	Citrate	M-H	7.1	↑	NS	1.42	a
188.001	1.61	2-aminophenol sulfate	M-H	3.9	↑	< 0.001	8.19	b
194.045	3.17	N-Salicyluric acid	M-H	4.2	↓	NS	− 1.48	c
181.049	3.29	Dihydrocaffeic acid	M-H	4.7	↓	NS	− 1.92	b
261.006	3.65	Dihydrocaffeic acid-3-O-sulfate	M-H	4.8	↓	NS	− 4.34	b
263.102	3.84	Phenylacetylglutamine	M-H	4.3	↑	NS	1.16	b
245.012	3.93	Vanillin 4-sulfate	M-H	6.1	↓	NS	− 2.59	b
287.022	4.14	DHPV O-sulfate	M-H	4.8	↑	NS	1.73	b
345.154	4.89	Steroid glucuronide		3.7	↑	NS	3.33	c
269.149	4.91	Unknown		6.7	↑	< 0.001	3.89	c
329.16	5.63	Steroid glucuronide		3.6	↓	0.012	− 6.50	c
367.157	6.48	Unidentified steroid		5.1	↑	NS	4.75	c

*FC* fold change; *m/z* mass–charge ratio; *RT* retention time; *VIP* variable importance in projection score ^†^Level of metabolite identification: (a) identified compound; (b) putatively annotated compound; (c) putatively characterised compound class;

and (d) unknown ^‡^False discovery rate (FDR) adjusted p-value (or q-value).

**Figure 2:**
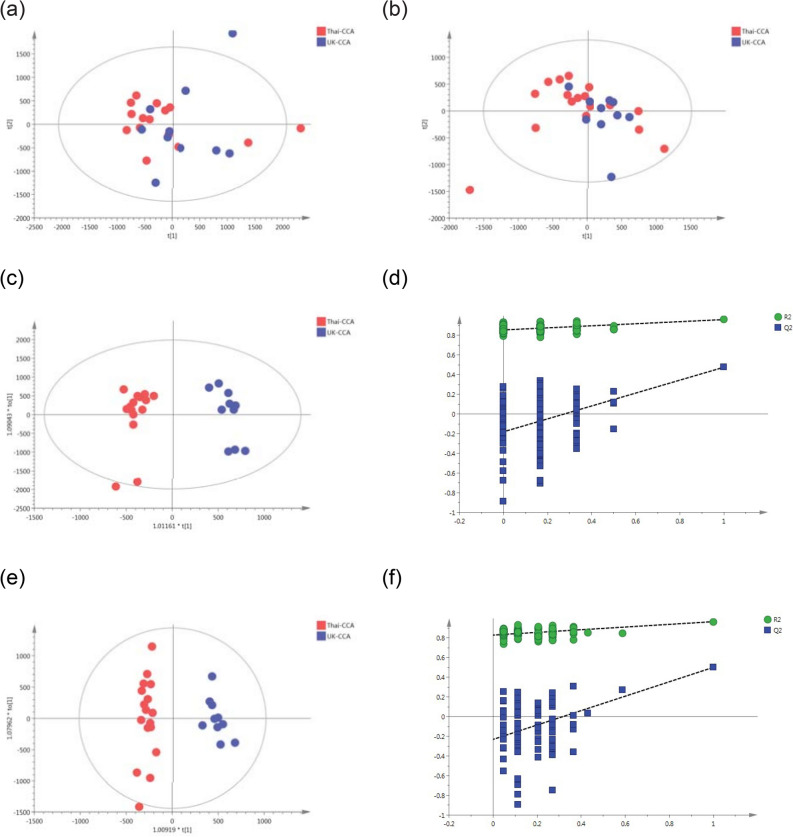
PCA scores plots for (**a**) positive and (**b**) negative ion mode data of CCA patients from Thailand and United Kingdom. OPLS-DA scores plots showing group separation for both (**c**) positive and (**d**) negative ion mode data and the corresponding permutation tests for (**e**) positive and (**f**) negative ion mode data. (**a**) PCA scores plot—positive mode R2X = 22% and Q2X =  − 2% from 2 PC model; (**b**) PCA scores plot—negative mode R2X = 27% and Q2X =  − 4% from 2 PC model; (**c**) OPLS-DA scores plot—positive mode R2Y = 96%, R2X = 20% and Q2Y = 48% , from one predictive and one orthogonal components model; (**d**) OPLS-DA scores plot—negative mode R2Y = 97%, R2X = 21% and Q2Y = 50%, from one predictive and one orthogonal components model; (**e**) Permutation test—positive mode CV-ANOVA = 0.009; (**f**) Permutation test—negative mode CV-ANOVA = 0.003.

The supervised orthogonal projections to latent structures discriminant analysis (OPLS-DA) model comparing the metabolic signature from patients with biliary carcinoma between the two populations was also discriminant with Q^2^ Y value of 48% in ESI+ and 50% in ESI– and significant ANOVA of cross‐validated residuals (CV‐ANOVA) *p *value= 0.009 and 0.003, respectively (Figure 2, Table 4).

**Table 4:** Altered metabolites between participants with cholangiocarcinoma from United Kingdom (*n* = 10) and Thailand (*n* = 14).

*m/z*	RT	Tentative assignment	Adduct	VIP	p value^‡^	FC	Identification^†^
162.113	0.50	L-carnitine (C1)	M + H	4.5	NS	2.14	a
114.065	0.52	Creatinine	M + H	6.5	NS	− 1.18	b
144.102	0.58	Proline betaine	M + H	4.6	NS	1.22	b
229.119	0.59	Hydroxyprolyl-Proline	M + H	5.6	NS	1.17	b
160.133	0.70	Unknown		5.0	NS	3.41	d
204.125	0.83	L-Acetylcarnitine (C2)	M + H	10.8	NS	1.13	a
166.073	1.17	7-Methylguanine	M + H	4.5	NS	− 1.35	b
150.078	1.21	N-methyladenine	M + H	4.7	0.014	− 1.88	
153.066	2.20	N-methyl-2-pyridone-5-carboxamide	M + H	5.2	NS	− 1.31	b
265.117	3.93	Unknown		4.9	0.002	2.81	d
279.132	4.44	Dipeptide C_14_H_18_N_2_O_4_	M + H	4.8	0.004	7.76	c
287.207	4.93	Acylcarnitine (C8:1)	M + H	6.0	< 0.001	4.1	b
310.202	5.35	Acylcarnitine (C10:3)	M + H	8.0	0.01	3.73	b
314.233	5.78	Decenoylcarnitine (C10:1)	M + H	4.7	NS	1.01	b
135.030	0.50	Threonate/Erythronate	M-H	3.3	NS	− 1.34	b
191.018	0.65	Isocitrate	M-H	4.6	NS	1.37	a
243.061	0.85	Uridine/pseudouridine	M-H	3.7	NS	1.44	b
191.018	1.07	Citrate	M-H	5.3	NS	− 1.44	a
260.022	1.79	Unknown	M-H	3.2	NS	− 1.13	d
216.032	1.92	Tyramine-O-sulfate	M-H	3.3	NS	3.48	b
277.003	3.64	Unknown	M-H	4.1	0.050	6.12	b
263.102	3.84	Phenylacetylglutamine	M-H	6.5	NS	−1.04	b
243.134	4.18	Hydroxyprolyl-Isoleucine	M-H	4.3	0.041	2.29	b
269.149	4.91	Unknown		5.6	< 0.001	2.76	c
329.16	5.63	Unidentified steroid	M-H	3.3	NS	9.74	c
331.175	5.99	Unidentified steroid	M-H	3.4	NS	1.10	c
514.283	6.09	Taurocholic acid	M-H	4.4	NS	2.43	b
464.301	6.24	Glycocholic acid	M-H	3.2	NS	−2.32	a

FC = fold change; RT = retention time; VIP = variable importance in projection score ^†^Level of metabolite identification: (a) identified compound; (b) putatively annotated compound; (c) putatively characterised compound class; and (d) unknown ^‡^False discovery rate adjusted p-value (or q-value).

### Univariate analysis of discriminatory metabolites

**Figure 3:**
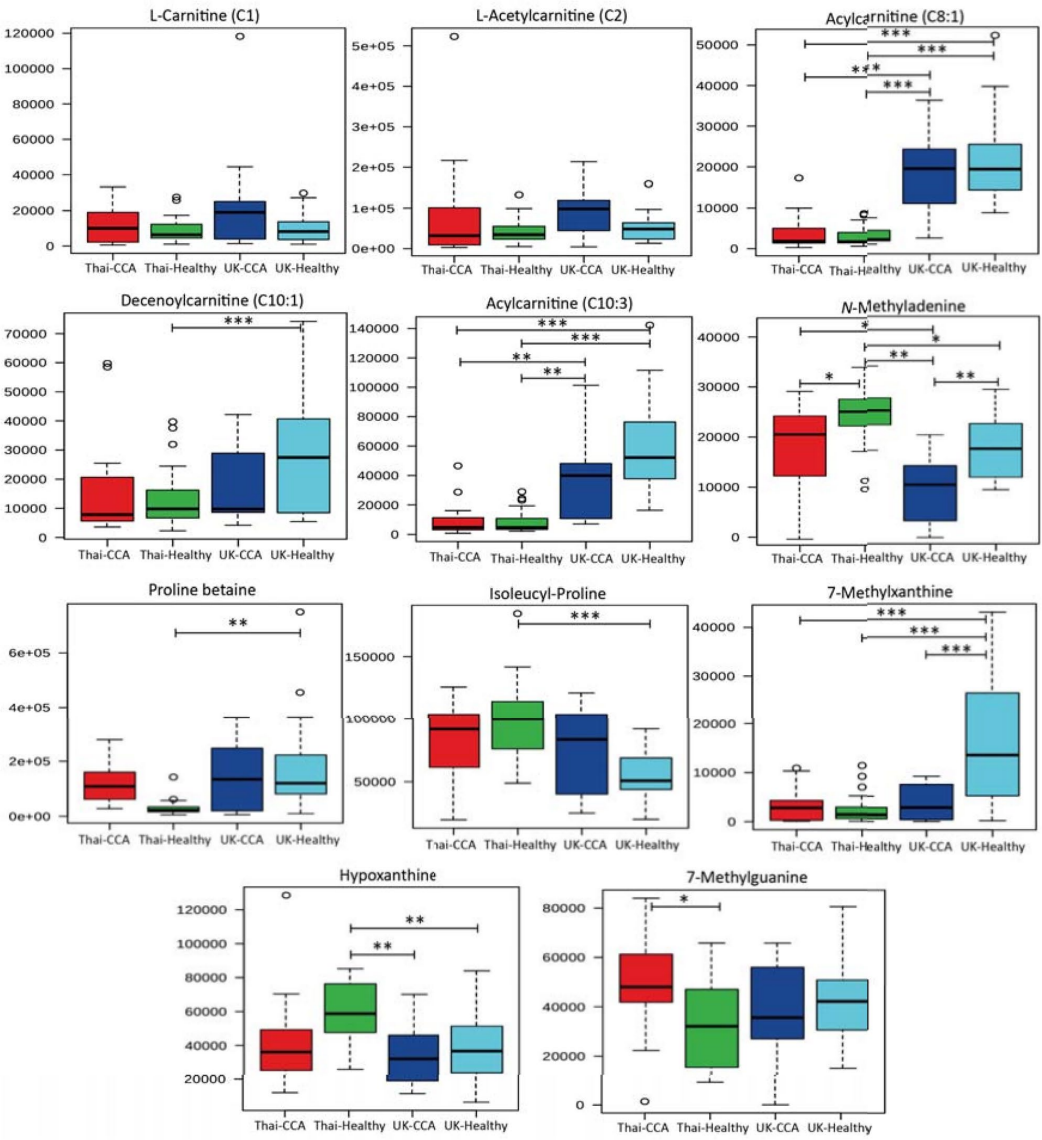
Box and whisker plots of altered urinary compounds among the study groups. Key: *p 0.05, **p 0.01 and ***p 0.001. Y-axis = relative intensity.

**Figure 4:**
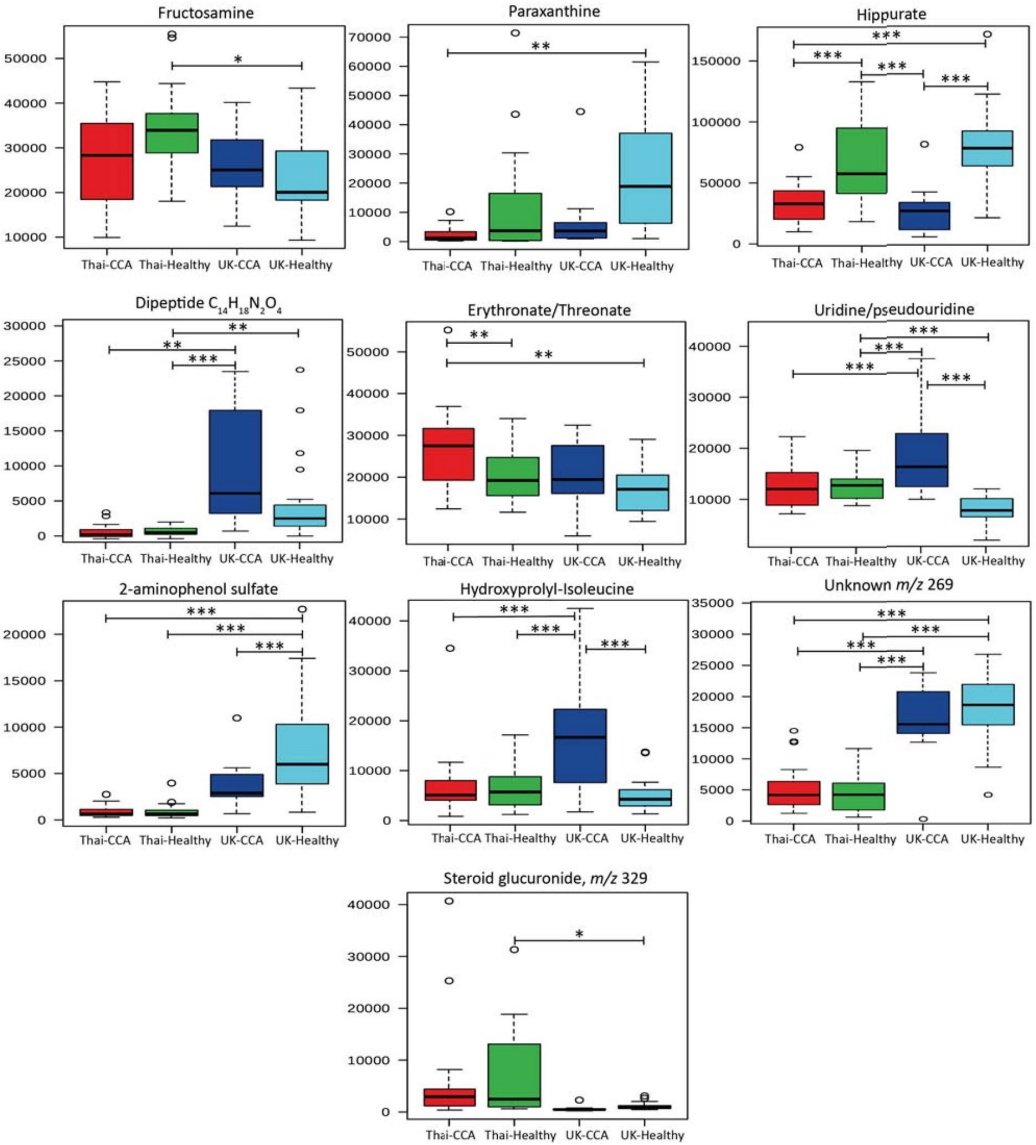
Box and whisker plots of altered urinary compounds among the study group. Key: *p 0.05, **p 0.01 and ***p 0.001. Y-axis = relative intensity.

The urinary levels of significantly discriminant metabolites (*p* < 0.05) generated from the previous two OPLS-DA models comparing the metabolic profiles between the two distinct populations in healthy and disease states are illustrated in Fig. 3 and 4. Statistical significance testing (presented in the whisker-box plots) was performed using ANOVA with post-hoc test to account for false discovery rate.

## Discussion

MS-based metabolic profiling unveiled population differences between the urine metabolome from the Thai and British cohorts. Urinary biomarkers of the dietary intake of citrus fruits (proline betaine)^14^, caffeine (7-methylxanthine and paraxanthine)^15^ and whole grains (2-aminophenol sulfate)^16^ were mostly associated with healthy Western participants. Additionally, the relative levels of metabolites related to proline-dipeptide metabolism (hydroxyprolyl-isoleucine and tyrosyl-proline/phenylalanyl-hydroxyproline) were significantly (ANOVA q-value <0.001) more abundant in the urine metabolite profiles from Western patients with CCA, compared to all other groups. Proline-containing dipeptide loss in urine is most likely related to protein catabolism pathways^17^, unlike dipeptides containing other amino acids (such as glutamate), which have been reported to be dysregulated in a variety of liver conditions^18,19^. Of note, for example, metabonomic analysis of sera in published studies has revealed that patterns of circulating glutamate-dipeptides could discriminate between different liver disease etiologies and were proposed as markers of glutathione depletion and increased oxidative stress^19^.

Medium-chain acylated carnitine species (C8:1, C10:1 and C10:3) were significantly more abundant in urine samples from the entire UK cohort, whereas the urinary excretion pattern of carnitine with short-chain acylated groups, L-carnitine (C1) and L-acetylcarnitine (C2), was uniform in the two populations, with relatively elevated concentrations in participants with biliary tumors. Elevation in acylated carnitines with 8 and 10 carbon atoms have been suggested to be associated with obesity^20^. A targeted acylcarnitine MS-assay of lean versus “healthy” obese individuals identified a metabolic pattern of human adiposity, which was characterized by imbalance in free fatty acid metabolism, overload of branched-chain amino acid (BCAA) catabolism and accumulation of acylated carnitines in serum **(*e.g.* C8:1 level in obese= 0.28 μM and in lean= 0.18 μM, *p*= 0.0006)^21^.

Obesity-induced insulin resistance, conventionally measured by the homoeostasis model assessment (HOMA) index, was also prevalent among the obese cohort with a 2.3-fold increase, compared to lean controls (*p*<0.0001). Dysregulation in lipids (including fatty acid and acylated carnitine species) and BCAAs are a common theme across metabolic profiling studies, as described in a recent systematic review and meta-analysis (46 publications) evaluating metabolite markers of pre-diabetes and type 2 diabetes^22^.

The interplay between lipids and BCAAs is proposed to modulate and promote the progression of insulin resistance in obesity. Reduction in BCAA catabolic activity in adipose tissue, most likely through the inhibition of peroxisome proliferator—activated receptor-*γ* (PPAR-*γ*) activity, overnutrition, host genetics and/or the gut microbiome, leads to an increase in the circulating BCAA levels^23^. Subsequently, the expanded circulating BCAA pool spills into skeletal muscle and liver tissue, promoting acylcarnitine generation from the transamination and oxidative decarboxylation products of BCAA (acyl-CoA derivatives). This may contribute to further metabolic adaptations observed in obesity and prediabetes states, such as inhibition of active fatty acid oxidation, mitochondrial stress and impaired insulin sensitivity, and ultimately impaired glucose homeostasis^23^. It was not possible to evaluate the association between obesity and acylcarnitine concentration for this cohort because continuous data on BMI were not available. However, rural residents with primary education in Thailand (typically farmers, with representative characteristics to the study cohort) tend to have higher lean mass compared to urban residents^24^.

It is generally held that cancer cells outstrip normal energy metabolism owing to increased energy requirements (the Warburg hypothesis)^25^. Thus, the Warburg effect in cancer cells allows unrestrained glycolysis leading to an up-regulation in compensatory pathways that feed into the citric acid cycle, specifically, increased oxidation of BCAA and fatty acids^25^. The short-chain acylated carnitines are derived from alternative energy sources, the BCAA: leucine, isoleucine, and valine^26^. During times of protein catabolism, as in cancer cachexia, the BCAA concentrations can increase^26^. Thus, the metabolic changes in the acylated carnitines and in BCAA metabolism found in CCA in our study are not specific to this tumour alone, but are present in other malignancies^27^. Perturbations in acylated carnitines and BCAA have been found also been found in hepatocellular carcinoma, lung cancer and breast cancer metabolomic studies^27^^.^. However, further study of acylated carnitine metabolism in larger cohorts may hold promise for potential biomarker development.

The urinary output of hippurate and a nucleoside compound putatively assigned as *N*-methyladenine was significantly lower in the urine metabolome of individuals with biliary carcinoma, regardless of the country from which they emanated. In the current study and in the literature, urinary hippurate has been found consistently to be positively related to the “healthy” metabotype and is inversely correlated with BMI^28^. Greater urinary excretion of methyladenine has been demonstrated to be related to cigarette smoke exposure^29^. Yet, another plausible hypothesis is that the gut microbiota may also influence urine methyladenine excretion.

A recent *in vivo* study investigated the effect of manipulating bacterial communities on the urine metabolome of ataxia telangiectasia mutated (Atm) knockout mice, which have an increased susceptibility to leukaemia/lymphoma^30^. Atm-deficient and wild type mice with a restricted intestinal microbial diversity showed a corresponding increase in metabolites reported to exert tumor-suppressor properties (including 3-methyladenine, kynurenic acid and 3-methylbutyrolactone) in urine, compared to mice with conventional gut microbiota^30^.

Elevated relative concentrations of uridine or pseudouridine appears to be specific to the metabolic signature of CCA in the Western cohort. This nucleoside has also been shown to be relatively elevated in the urinary metabolic profiles from Western participants with pancreatic carcinoma and non-malignant biliary strictures, suggesting a possible perturbation across the nucleotide metabolic network resulting from an increase in the rate of RNA turnover during precancerous and cancerous transformations^31,32^. Metabolic reprogramming in the mitochondrial matrix (including nucleotide biosynthesis) provides critical biosynthetic routes to support cell proliferation and is emerging as a potential cancer therapeutic target. This observation may provide mechanistic insight into differences in mitochondrial energy production between CCA subtypes (sporadic *vs.* liver fluke-related CCA)^33^.

Higher levels of 7-methylguanine was observed in patients with biliary carcinoma, compared to the control group in the cohort from Thailand. However, no statistically significant difference was observed amongst the two populations (Thailand *vs.* UK). The presence of such methylated purine derivatives in the urine has been found to be an indicator of endogenous and exogenous background of DNA methylation and pathologies affecting purine metabolism, such as gout^34,35^. In Thailand, the bioaccumulation of nitrogen species in the water reservoirs and food chain (such as fermented pork and fermented fish) is believed to play an important carcinogenic role, along the parasitic infestation, in the pathogenesis of hepatobiliary carcinoma^36,37^. A metabolite tentatively identified as erythronate/threonate was associated with the urinary metabolic signature of liver fluke associated-CCA. The variation in concentrations of erythronate/threonate (an end-product of ascorbic acid degradation) in biofluids, might be related to differences in dietary habits, drug use and/or underlying comorbidities (such as hypertension)^38,39^.

## Conclusion

Further understanding of intrinsic and extrinsic factors that affect the metabolic status of the human urine metabolome is required to gain a better understanding of metabolic adaptations and resulting metabolic end product “metabolites” in health and disease^40^. Herein, population-specific differences between the two ethnic groups were found to be considerably influenced by factors related to variation in dietary habits and body composition. This study was limited by the small numbers of study participants, owing to the rarity of the tumour and the difficulty in sample collection, and although no diagnostic biomarker for CCA was evident in this study population, the perturbation in acylated carnitines is worthy of further study in larger cohorts.

The observations derived from this small dataset point towards potential host-gut microbiota metabolic interactions in CCA carcinogenesis, regardless of the underlying etiology. Whereas, metabolic markers implicated in nucleotide metabolism were associated with the sporadic type of hepatobiliary disease most common in Western countries, possibly indicating increase flux through energy-providing pathways in the mitochondrial matrix to support cellular proliferation.

## Data Availability

Available from Larry Koomson, Department of Metabolism, Digestion and Reproduction, Imperial College London, London, W2 INY, United Kingdom (l.koomson@imperial.ac.uk).

## Abbreviations

ANOVA: Analysis of variance
BCAA: Branched chain amino acids
CCA: Cholangiocarcinoma
CID: Collision-induced dissociation
CT: Computed tomography
CV‐ANOVA: ANOVA of cross‐validated residuals
DDA: Data-dependent acquisition
ESI: Electrospray ionization
ESI −: Electrospray ionisation negative mode
ESI +: Electro spray ionisation positive mode
LC–MS: Liquid chromatography mass spectroscopy
MRI: Magnetic resonance imaging
NMR: Nuclear magnetic resonance
OPLS-DA: Orthogonal projections to latent structures discriminant analysis
QC: Quality control
RP: Reverse phase
TOF: Time of flight
UPLC: Ultra performance liquid chromatography

## Declaration

This paper is based on a chapter in the thesis of the first author, Munich Alsaleh—to which all author authors had academic contribution or supervision^41^. Professor Puangrat Yongvanit died during preparation of this manuscript. She contributed substantially to the work. Approval has been obtained from her legal representatives for her inclusion in this manuscript as an author. Further details are available from Professor Ross Andrews, Cholangiocarcinoma Research Centre, Faculty of Medicine, Khon Kaen University, Khon Kaen 40002, Thailand (rhandrews@gmail.com). Professor Roger Williams also died during manuscript preparation, but contributed substantially to the work. Approval has been obtained from his legal representatives for his inclusion in this manuscript as an author. Further details are available from Natalie Day at The Institute of Hepatology, 111 Coldharbour Lane, London SE5 9NT (n.day@researchinliver.org.uk).
